# Reassessing the Composition of Hybrid Orbitals in Contemporary VB Calculations

**DOI:** 10.1021/acs.jpca.3c01857

**Published:** 2023-05-31

**Authors:** David L. Cooper, Fabio E. Penotti, Peter B. Karadakov

**Affiliations:** † Department of Chemistry, University of Liverpool, Liverpool L69 7ZD, U.K.; ‡ Consiglio Nazionale delle Ricerche, Istituto di Scienze e Tecnologie Chimiche “Giulio Natta”, Via Golgi 19, I-20133 Milano (MI), Italy; § Department of Chemistry, University of York, Heslington, York YO10 5DD, U.K.

## Abstract

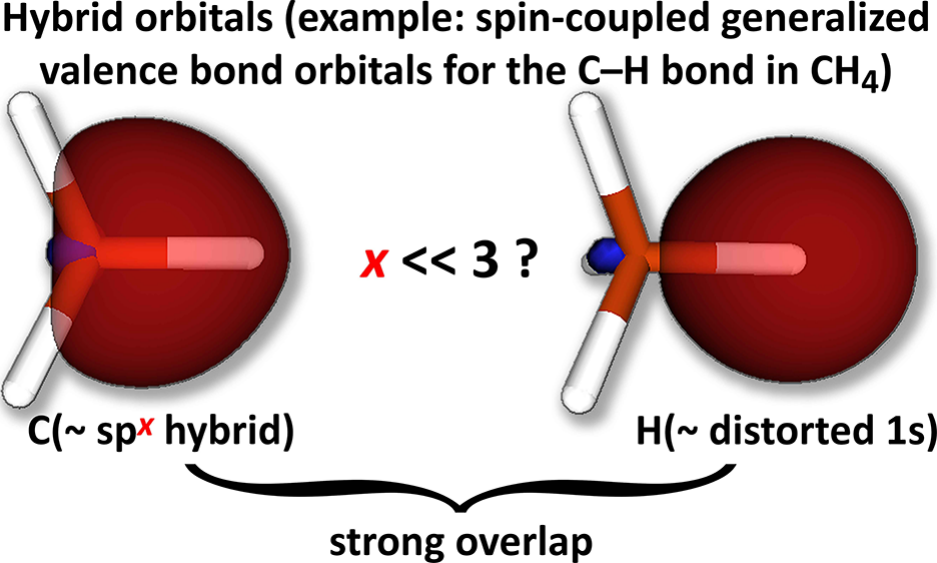

Large variations in the ratios between the p and s components of individual hybrid orbitals that have been observed in contemporary ab initio VB calculations are reassessed, and links are established to specific energy terms that drive bond formation. It is demonstrated that the ratios between the p and s components for individual hybrid orbitals are not indicative of the overall hybridization status of the relevant atom, which exhibits only relatively small variations with the level of theory, irrespective of whether or not non-dynamical and dynamical electron correlation effects are accounted for. An alternative orbital representation that turns out to be far more consistent with the overall hybridization of the relevant atom is examined. The chosen test cases, which can be compared with the classical sp^3^, sp^2^, and sp hybridization models for a central carbon atom, are CH_4_ (T_*d*_), trigonal CH_3_ (D_3*h*_), and triplet CH_2_ distorted from its ground state geometry so as to be linear (D_∞*h*_).

## 1 Introduction

Conventional notions of hybrid atomic orbitals (HAOs), such as those invoked in the familiar models of sp^3^, sp^2^, and sp hybridization for the carbon atom, remain deeply embedded in chemical thinking. Hybrid orbitals have been observed for wide ranges of molecules in fully variational optimizations of modern ab initio valence bond wave functions; in the case of methane, for example, four equivalent tetrahedrally oriented carbon-based sp^*x*^-like hybrids are obtained directly in spin-coupled generalized valence bond (SCGVB) calculations,^1^ without the imposition of any constraints or preconceptions. On the other hand, whereas the four sp^3^ HAOs involved in the traditional VB description of CH_4_ were envisaged by Pauling and Slater as being mutually orthogonal,^2,3^ the corresponding fully optimized sp^*x*^-like SCGVB hybrids have substantial overlap. Consistent with the expected contraction of HAOs on bond formation, such nonorthogonality is associated with enhanced 2s character^4^ in each of the four equivalent sp^*x*^-like SCGVB hybrids relative to the classical sp^3^ hybrid orbitals. Following the notation introduced by Xu and Dunning,^5,6^ it proves useful in what follows to use *h*_*n*p/*n*s_ to denote the value of *x* in sp^*x*^, i.e., the p/s ratio, so that *h*_2p/2s_ = 3 for the orthogonal classical sp^3^ HAOs of methane, whereas *h*_2p/2s_ < 3 for the corresponding nonorthogonal sp^*x*^-like SCGVB hybrids.

Shaik et al.^7^ have shown how *h*_*n*p/*n*s_ values for linear, trigonal, or tetrahedral HAOs can be deduced from the overlaps between these orbitals, and they used a range of ab initio valence bond self-consistent field (VBSCF) results to support an appealing interpretation of the reduction of *h*_*n*p/*n*s_ from the corresponding values for classical HAOs, focusing on the balance between promotion and bond energies. In terms of overall energy changes, we can of course think of the formation of the ‘closed shell’ electronic ground state of CH_4_ from ground state C(^3^P) and H(^2^S) atoms as a two-step process: first there is the promotion energy from C(^3^P) to C(^5^S) but this is more than compensated in the second step by the energy gain associated with the formation of the four C–H bonds. According to Shaik et al.,^7^ lower *h*_2p/2s_ values, corresponding to enhanced 2s character, have the straightforward consequence that the promotion energy required to achieve maximum bonding is smaller. Thus, in general terms, a larger overlap between the HAOs on the central atom for a given geometric arrangement corresponds to a smaller *h*_*n*p/*n*s_ value and thus to a reduction in the required promotion energy.

An attractive scheme for evaluating *h*_*n*p/*n*s_ values and related quantities has been used by Xu and Dunning^5,6^ to examine the parentage of individual SCGVB orbitals. In essence, their approach involves projecting atomic *n*s and *n*p functions onto the orbital being analyzed. Comparing the corresponding *h*_*n*p/*n*s_ values for orbitals from ‘full’ SCGVB treatments with those from certain constrained SCGVB calculations revealed rather large variations, with the range in the case of methane, for example, being from ca. 0.6 to nearly the classical value of 3.^5^ Given that the energy differences between these wave functions^5^ were fairly modest relative to the full C(^3^P) to C(^5^S) excitation energy of 153.7 m*E*_h_ (96.45 kcal/mol), it seems unrealistic to try to attribute such dramatic changes in *h*_2p/2s_ values to differences in the notional promotion energy of the C atom.

The main aims of the present study are to examine further some of the wide variations in *h*_*n*p/*n*s_ values for individual SCGVB orbitals that are revealed by the mode of analysis of Xu and Dunning,^5,6^ to assess the variations in the corresponding hybridization status of the central atom, to explore an alternative orbital representation that turns out to be more consistent with the overall hybridization of the central atom, and to try to establish links to specific energy terms that drive bond formation. As suitable test cases, which we may compare with classical sp^3^, sp^2^, and sp hybridization of a central carbon atom, we employ CH_4_ (T_*d*_), trigonal CH_3_ (D_3*h*_), and triplet CH_2_ distorted from its ground state geometry so as to be linear (D_∞*h*_).

## 2 Theoretical Background and Computational Details

The SCGVB wave functions considered in the present study take the form:^8^
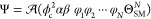
1in which ϕ_c_ is a doubly occupied core orbital, the φ_μ_ are the singly occupied nonorthogonal SCGVB orbitals for the *N* valence electrons, and Θ_SM_^*N*^ is the total spin function for the valence electrons. All the core and valence orbitals, which were expanded in the full molecular basis set, were optimized simultaneously with the total spin function, which was expanded in the full space of Kotani functions^9^ for *N* electrons coupled to total spin quantum number S and projection M. In addition to these ‘full’ SCGVB calculations, we also optimized SCGVB(PP) wave functions, with Θ_SM_^*N*^ restricted to the perfect pairing (PP) mode of spin coupling and also, in addition, with strong orthogonality (SO) of the valence orbitals, such that the SCGVB(PP/SO) orbitals in different pairs are constrained to be orthogonal to one another.

The key feature that makes the atomic orbital composition approach of Xu and Dunning^5,6^ for cc-pVnZ and aug-cc-pVnZ basis sets so straightforward is that the first generally contracted basis functions in the cc-pVnZ basis set for each atomic symmetry are the occupied atomic orbitals (φ_*n*s_^atom^, φ_*n*p_^atom^, ···).^10^ Central to the scheme is the evaluation of matrix elements, such as ⟨φ_*n*s_^atom^ | *Q* | φ_*n*s_^atom^⟩, in which *Q* is a quantity with the dimensions of an electron density and φ_*n*s_^atom^ is the relevant generally contracted basis function. In the case of an individual SCGVB orbital, φ_μ_, the quantity *Q* to be analyzed is |φ_μ_⟩⟨φ_μ_|, so that:
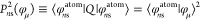
2with an analogous definition for *P*_*n*p_^2^(φ_μ_). The total contribution *P*_*n*s+*n*p_^2^(φ_μ_) of these *n*s and *n*p atomic orbitals is then given by:
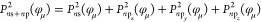
3and the fractions of *n*s and *n*p atomic orbital character by:
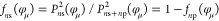
4a
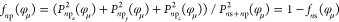
4bAccordingly, the hybridization ratio *h*_*n*p/*n*s_(φ_μ_) is given by:
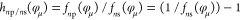
5

Furthermore, using the expansion of the active-space electron density ρ in terms of the SCGVB orbitals, i.e.,
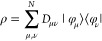
6in which *D*_μν_ is an element of the corresponding normalized spinless 1-particle density matrix, we may define:
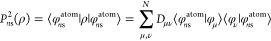
7with an analogous expression for *P*_*n*p_^2^(ρ). We may thus straightforwardly obtain values of *f*_*n*s_(ρ), *f*_*n*p_(ρ), and *h*_*n*p/*n*s_(ρ). The occurrence of off-diagonal terms in the expression for the active-space electron density ρ (eq 6) is linked to the nonorthogonality of the SCGVB orbitals, and it has the consequence that the p/s ratio for ρ is likely to differ from those for individual C-based hybrids.

In the present study, we also use an implementation of Cioslowski’s QTAIM-based isopycnic transformation^11^ to generate sets of localized valence natural orbitals (LNOs) ψ_*i*_^LNO^ that reproduce the active-space electron densities. For the subsequent atomic orbital composition analysis, we may write for example:
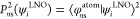
8and using the expansion of the valence electron density ρ in terms of such LNOs, i.e.,
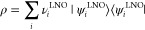
9in which the ν_*i*_^LNO^ are the corresponding occupation numbers, we obtain for example:
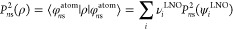
10Of course, provided that we are analyzing the same electron density, the resulting set of values of *f*_*n*s_(ρ), *f*_*n*p_(ρ), and *h*_*n*p/*n*s_(ρ) must be the same (to usual numerical precision) whether we expand ρ in terms of SCGVB orbitals, LNOs, or orthonormal canonical natural orbitals. Note that although such LNOs need not be orthogonal to one another, the expression for ρ (eq 9) involves only diagonal terms. (Amongst various alternatives to the generation of LNOs using the isopycnic transformation, we could have pursued the real-space adaptive natural density partitioning approach introduced by Francisco et al.^12^)

All of the electronic structure calculations were performed with the MOLPRO package^13−15^ using aug-cc-pVQZ basis sets and reoptimizing the C–H bond length *r*_CH_ at each level of theory. Visual depictions of various orbitals were produced from Virtual Reality Markup Language (VRML) files generated with Molden,^16^ using the same isovalue (±0.1) throughout. Quantum theory of atoms in molecule (QTAIM) analysis^17^ of total densities was carried out using AIMAll,^18^ with all other analysis making use of our own codes.

## 3 Results and Discussion

We consider first the results for the ground state of CH_4_ (T_d_) at the restricted Hartree-Fock (RHF), SCGVB(PP/SO), SCGVB(PP), ‘full’ SCGVB and CASSCF(8,8) levels, in which the latter denotes a complete active space self-consistent field construction with an active space of 8 electrons in 8 orbitals. These wave function calculations, for which the optimized values of *r*_CH_ and the corresponding total energies *E*_total_ are listed in Table 1, are in effect the same as those reported by Xu and Dunning,^5^ enabling direct comparison of the two sets of results. To assess the impact of dynamical electron correlation, we also consider CCSD(fc) results at the geometry from a full CCSD(T) optimization and also values derived from CASSCF(8,8)+1+2 internally-contracted multireference configuration interaction (MRCI) calculations.

**Table 1:** Total Energies (*E*_total_) and Relative Energies (Δ*E*_total_), Optimized C–H Bond Lengths (*r*_CH_), and Various p/s Ratios (*h*_2p/2s_) for CH_4_ (T_*d*_), CH_3_ (D_3*h*_), and Linear Triplet CH_2_ (D_∞*h*_)

	level of theory	*r*_CH_ (Å)	*E*_total_ (*E*_h_)	Δ*E*_total_ (kcal/mol)	*h*_2p/2s_		
					φ_1_	σ valence density	ψ_1_^LNO^
CH_4_	RHF	1.08154	–40.216346	0		2.715	
	SCGVB(PP/SO)	1.10021	–40.278165	–38.8	2.971	2.705	2.703
	SCGVB(PP)	1.10162	–40.279675	–39.7	1.582	2.690	2.689
	SCGVB	1.10195	–40.282402	–41.5	0.570	2.692	2.690
	CASSCF(8,8)	1.10074	–40.299864	–52.4		2.698	2.697
	MRCI	1.08854	–40.445412	–143.7		2.706	2.703
	CCSD(T)	1.08599	–40.482519	–167.0			
	CCSD(fc)^a^					2.710	2.706
CH_3_	RHF	1.06917	–39.576026	0		1.865	
	SCGVB(PP/SO)	1.08537	–39.620835	–28.1	1.978	1.861	1.861
	SCGVB(PP)	1.08701	–39.622313	–29.0	0.897	1.851	1.851
	SCGVB	1.08968	–39.627190	–32.1	0.532	1.852	1.852
	CASSCF(7,7)	1.09050	–39.635671	–37.4		1.854	1.855
	MRCI	1.07824	–39.768076	–120.5			
CH_2_	RHF	1.05408	–38.917337	0		1.003	
	SCGVB(PP/SO)	1.06710	–38.944351	–17.0	0.999	1.005	1.005
	SCGVB(PP)	1.06922	–38.946050	–18.0	0.259	0.997	0.997
	SCGVB	1.07677	–38.958850	–26.0	0.564	1.003	1.003
	CASSCF(6,6)	1.07775	–38.962432	–28.3		1.003	1.004

a Using *r*_CH_ from full CCSD(T).

**Figure 1:**
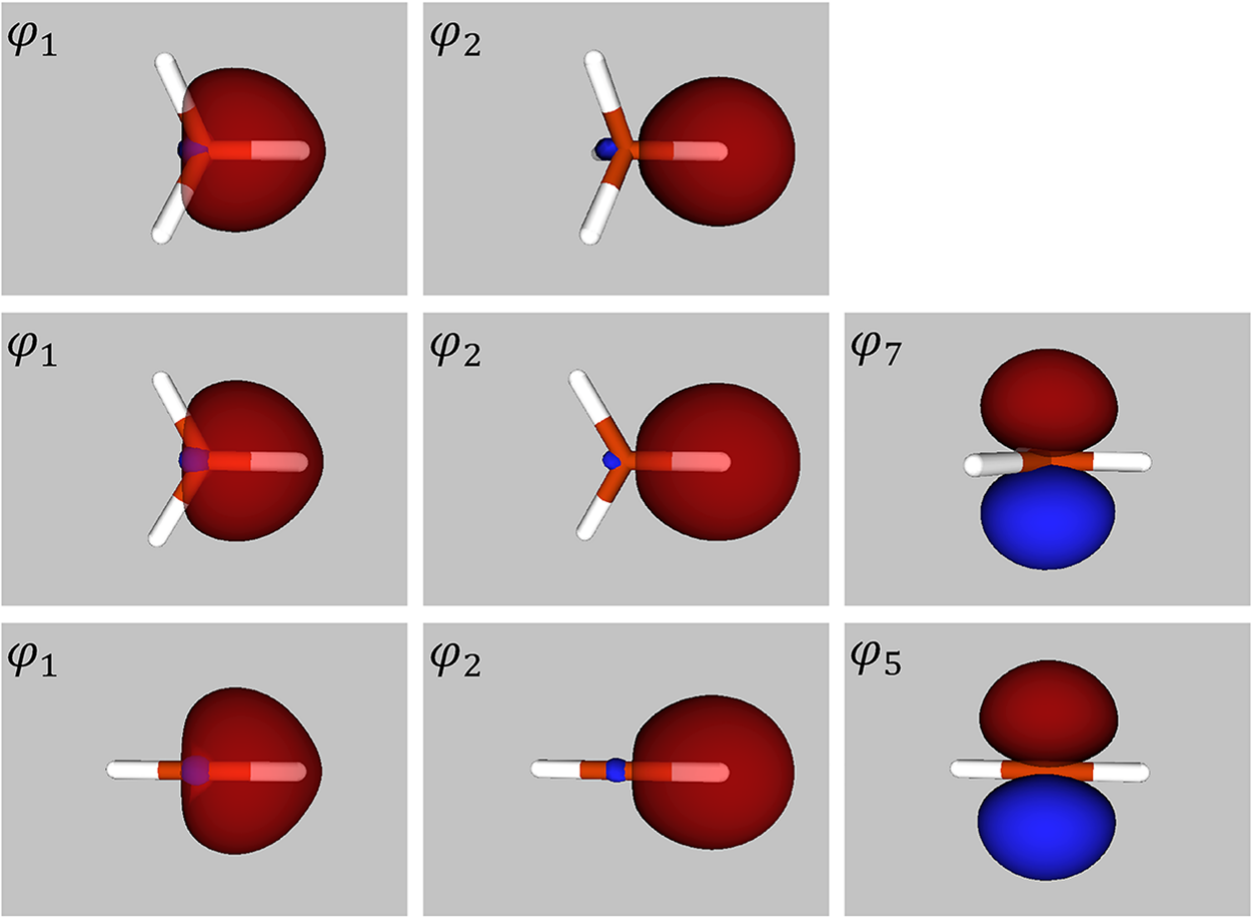
Depictions of symmetry-unique SCGVB orbitals for CH_4_ (top row), CH_3_ (middle row), and linear triplet CH_2_ (bottom row).

As has been well established,^1,5,19^ the fully optimized SCGVB valence orbitals for CH_4_ spontaneously take the form of four equivalent tetrahedrally oriented C-based sp^*x*^-like hybrids, each of which overlaps an orbital on the corresponding H center. These C-based sp^*x*^-like hybrids arise in these calculations without any preconceptions. As can clearly be seen from visual depictions of symmetry-unique orbitals φ_1_ and φ_2_ (see top row of Figure 1), the C-based orbital φ_1_ is distorted toward and partially delocalized onto the H center, and vice versa for the H-based orbital φ_2_. The ⟨φ_1_ | φ_2_⟩ overlap is 0.699. The corresponding orbital pairs directed along the other C–H bonds are (φ_3_, φ_4_), (φ_5_, φ_6_), and (φ_7_, φ_8_), with the odd-numbered orbitals being the C-based hybrids and the even-numbered ones being the corresponding H-based orbitals. As is to be expected, by far the largest contributor to the total spin function is the perfect pairing mode of spin coupling, which has a weight of 0.9067 in the Kotani basis.

Unlike the sp^3^ HAOs that were invoked in the classical VB description of CH_4_,^2,3^ the SCGVB sp^*x*^-like hybrids are not orthogonal to one another, with the symmetry-unique overlap between different C-based orbitals, ⟨φ_1_ | φ_3_⟩, being 0.512. We find that restricting the total spin function to the perfect pairing mode leads to a significant increase in the ‘in-bond’ overlap ⟨φ_1_ | φ_2_⟩, to 0.827, and to an even larger decrease in the overlap between different C-based orbitals, ⟨φ_1_ | φ_3_⟩, to 0.179, whereas also imposing the requirement of strong orthogonality leads to only a small further increase in ⟨φ_1_ | φ_2_⟩, to 0.831. Visual representations of these SCGVB(PP) and SCGVB(PP/SO) orbitals, which are available in the Supporting Information, turn out to be rather similar to those from the ‘full’ SCGVB calculation (Figure 1). It is not surprising that the changes in the 2p to 2s ratio from SCGVB to SCGVB(PP) to SCGVB(PP/SO) are difficult to discern from such pictures, bearing in mind just how similar to one another are the corresponding depictions in position space of classical sp, sp^2^, and sp^3^ hybrids.^20^

The energy penalties for imposing PP and PP/SO constraints on the SCGVB wave function for CH_4_ are relatively small, such that the SCGVB, SCGVB(PP), and SCGVB(PP/SO) calculations recover 79.1, 75.8, and 74.0%, respectively, of the predominantly non-dynamical electron correlation that is incorporated in the CASSCF(8,8) wave function. Accordingly, the energy lowering from SCGVB(PP/SO) to SCGVB(PP) is just 0.9 kcal/mol and that from SCGVB(PP) to SCGVB is only 1.7 kcal/mol. Nonetheless, as can be seen from Table 1 and as was found by Xu and Dunning,^5^ the corresponding changes to the value of *h*_2p/2s_ for the C-based orbital φ_1_ are rather dramatic, ranging from less than 0.6 at the ‘full’ SCGVB level to nearly 3.0 for SCGVB(PP/SO). It is important to note that such a large variation is not simply an artifact of using the atomic orbital composition approach of Xu and Dunning.^5,6^ Although the precise numbers were of course different, we found analogous behavior when using various alternative schemes, including Mulliken population analyses (see Table S1 in the Supporting Information) and Löwdin-style symmetric orthogonalization. It is also useful to note that the dramatic reductions in the value of *h*_2p/2s_(φ_1_) do not correspond to any decline in the extent to which φ_1_ can be considered to be a C-based hybrid: *P*_2s+2p_^2^(φ_1_) is, in fact, slightly higher at the SCGVB level than at the SCGVB(PP) or SCGVB(PP/SO) levels, with the values being 0.985, 0.978, and 0.972, respectively.

**Figure 2:**
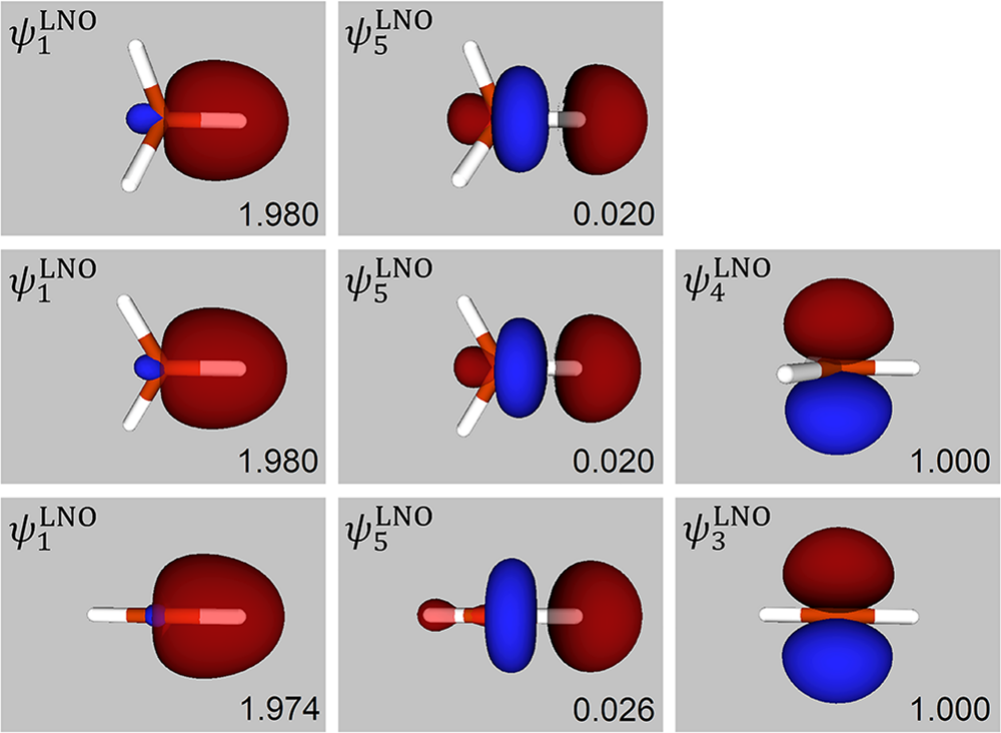
Depictions of symmetry-unique SCGVB valence LNOs for CH_4_ (top row), CH_3_ (middle row) and linear triplet CH_2_ (bottom row). Also shown are the corresponding occupation numbers, ν_*i*_^LNO^. The LNOs have been numbered in each case in order of decreasing ν_*i*_^LNO^.

As a first step toward identifying the extent to which the wide variation in *h*_2p/2s_(φ_1_) values could be indicative of changes to the overall hybridization status of the C atom, we used an implementation of Cioslowski’s isopycnic transformation^11^ to generate valence LNOs for the various post-RHF densities. We found that the descriptions are dominated in each case by four symmetry-equivalent orbitals ψ_1_^LNO^ – ψ_4_^LNO^ with high occupation numbers, namely 1.979, 1.980, 1.982, and 1.983 for CASSCF(8,8), SCGVB, SCGVB(PP) and SCGVB(PP/SO), respectively. These four LNOs are complemented by another four symmetry-equivalent orbitals, ψ_5_^LNO^ – ψ_8_^LNO^, with occupation numbers 0.021, 0.020, 0.018, and 0.016 for CASSCF(8,8), SCGVB, SCGVB(PP), and SCGVB(PP/SO), respectively. As can be seen from the top row of Figure 2, the form of ψ_1_^LNO^ for the SCGVB valence density corresponds to standard notions of a localized C–H bond, whereas ψ_5_^LNO^ is an antibonding combination. Note that although the valence LNOs need not be orthogonal to one another, the overlaps between them turn out to be small. For example, in the case of the ‘full’ SCGVB description of CH_4_, the largest of the symmetry-unique overlaps is just 0.029. Visual representations of the corresponding CASSCF(8,8), CCSD(fc), MRCI, SCGVB(PP) and SCGVB(PP/SO) LNOs, which are available in the Supporting Information, are very similar to those shown in Figure 2 for the ‘full’ SCGVB calculation.

We report in Table 1 our values of *h*_2p/2s_ for the total valence density from SCGVB(PP/SO), SCGVB(PP), ‘full’ SCGVB, CASSCF(8,8), MRCI and CCSD(fc) valence densities. We observe that all of these *h*_2p/2s_ values, including those for CCSD(fc) and MRCI, exhibit only rather small deviations from 2.7. One obvious conclusion is that the inclusion of dynamical electron correlation has relatively little effect on the overall hybridization status of the C atom. We also find that the corresponding values of *h*_2p/2s_ for ψ_1_^LNO^ (see Table 1) differ from those for the total valence density only in the third decimal place, so that the overall contributions from ψ_5_^LNO^ onwards are relatively insignificant in each case. The small variations in the *h*_2p/2s_ values for the total valence density, or in those for ψ_1_^LNO^, clearly suggest only very modest variations in the overall hybridization status of the C atom, regardless of the somewhat more dramatic behavior observed for *h*_2p/2s_(φ_1_) values. At least in this sense, the valence LNOs provide alternative orbital representations for CH_4_ that are more consistent with the overall hybridization status of the central C atom. Certainly, we must conclude for CH_4_ that p/s ratios for SCGVB orbitals, and likely VBSCF orbitals, do not provide a reasonable representation of the overall hybridization status of the carbon atom. Given that Shaik et al.^7^ deduced a p/s ratio of 1.76 for VBSCF orbitals by analyzing the overlaps between them, it could be worthwhile to show that the corresponding ratios for valence LNOs or for the valence electron density are somewhat higher.

Subtracting from the CASSCF(8,8)+1+2 internally-contracted MRCI energy of CH_4_ the corresponding CASSCF(7,7)+1+2 energy of CH_3_ (see Table 1) and the RHF energy of the H atom, we obtain a value for the CH_3_–H bond dissociation energy (BDE) of 111.3 kcal/mol. Use of the corresponding cluster-corrected energies (Davidson correction) yields 111.8 kcal/mol. These values are certainly consistent with the BDE derived from experiment, namely 112.5 kcal/mol,^21^ but it has been shown that still closer agreement requires the incorporation of core correlation effects and the use of larger basis sets.^21^ Even so, it should be clear whether we analyze the total valence density or ψ_1_^LNO^, and whether we do so using the current atomic orbital composition approach (see Table 1) or with Mulliken population analysis (see Table S1 in the Supporting Information), that we consistently obtain values of *h*_2p/2s_ for CH_4_ that are close to 2.7 at a level of theory that is more than sufficient to provide a good estimate of the CH_3_–H BDE.

Returning to the various SCGVB results, we find that the lowering of the total energy in the sequence from SCGVB(PP/SO) to SCGVB(PP) to SCGVB is accompanied not only by an increase in the symmetry-unique overlap between different C-based orbitals, ⟨φ_1_ | φ_3_⟩, from zero to 0.179 to 0.512 but also by a decrease in the ‘in-bond’ overlap ⟨φ_1_ | φ_2_⟩ from 0.831 to 0.827 to 0.699. Such behavior is somewhat counterintuitive because, in general terms, stronger bonds tend to have larger SCGVB orbital overlaps for the singlet-coupled pair that describes that bond, whereas the overlaps between orbitals in different singlet-coupled pairs tend to be destabilizing. Clearly it could be useful to identify a specific energy term that is linked to bond formation and that favors SCGVB over SCGVB(PP) over SCGVB(PP/SO).

Chemical bonding is, of course, a quantum chemical effect, with the physical origin of covalent bonding being linked to the interference of the fragment wave functions. The formation of conventional covalent bonds involves the lowering of the interatomic kinetic energy,^22−28^ due to electron delocalization in the bonding region, with the required interatomic kinetic energy lowering also driving intra-atomic contractions. Accordingly, it seems very worthwhile to investigate how the interatomic kinetic energy component for the C–H bonds in CH_4_ varies in the sequence from SCGVB(PP/SO) to SCGVB(PP) to SCGVB. Ideally, we should find values that vary monotonically with, but faster than, the corresponding total energies, *E*_total_. (We note that it has been reported for systems featuring charge-shift bonding that intra-atomic contractions are not accompanied by a reduction in the interatomic kinetic energy,^29,30^ but no such molecules are considered in the present study.)

As a straightforward estimate of the interatomic kinetic energy component for the four bonds, we restricted the double summation in the standard expression for the expectation value of the valence kinetic energy so to retain only those integrals that have one basis function on the C center and the other on H atoms. As an internal check, we confirmed that the same values (to usual numerical precision) are generated whether we express the valence density in terms of SCGVB orbitals or orthonormal canonical natural orbitals. The resulting quantities, which we label *K*_CH_^σ^, are listed in Table 2. We observe that *K*_CH_^σ^ does indeed vary monotonically with, but faster than, *E*_total_. We conclude from these values of Δ*K*_CH_^σ^ that the additional lowering of the interatomic kinetic energy due to incorporating increased 2s character into the C-based hybrids more than compensates for the consequences of the increased different-pair overlaps and reduced same-pair overlaps.

**Table 2:** Interatomic Kinetic Energy Components (*K*_CH_^σ^) and Differences in Total Energies (Δ*E*_total_) for Various Descriptions of CH_4_ (T_*d*_), CH_3_ (D_3*h*_), and Linear Triplet CH_2_ (D_∞*h*_)

	level of theory	*K*_CH_^σ^ (*E*_h_)	Δ*K*_CH_^σ^ (m*E*_h_)	Δ*E*_total_ (m*E*_h_)
CH_4_	SCGVB(PP/SO)	1.44443	0	0
	SCGVB(PP)	1.42336	–21.1	–1.5
	SCGVB	1.41404	–30.4	–4.2
CH_3_	SCGVB(PP/SO)	1.03740	0	0
	SCGVB(PP)	1.02186	–15.5	–1.5
	SCGVB	1.00743	–30.0	–6.4
CH_2_	SCGVB(PP/SO)	0.57797	0	0
	SCGVB(PP)	0.56894	–9.0	–1.7
	SCGVB	0.55367	–24.3	–14.5

It is important to ascertain the extent to which our general findings are specific to the notional sp^3^ case of CH_4_, or they apply more widely also to classical sp^2^ and sp hybridization. To this end, following the same general strategies as for CH_4_, we also examined trigonal doublet CH_3_ (D_3*h*_) and triplet CH_2_ distorted from its ground state geometry so as to be linear (D_∞*h*_). To enable future direct comparisons with the descriptions presented here, the various optimized *r*_CH_ values and total energies are recorded in Table 1. The SCGVB, SCGVB(PP), and SCGVB(PP/SO) calculations for CH_3_ recover 85.8, 77.6, and 75.1%, respectively, of the predominantly non-dynamical electron correlation incorporated in the CASSCF(7,7) wave function, with the analogous proportions for linear triplet CH_2_ being 92.1, 63.7, and 59.9%, respectively. Accordingly, the energy lowering for CH_3_ from SCGVB(PP/SO) to SCGVB(PP) is just 0.9 kcal/mol and that from SCGVB(PP) to SCGVB is only 3.1 kcal/mol, whereas the corresponding figures for linear triplet CH_2_ are 1.1 and 8.0 kcal/mol, respectively. Such values suggest that changes to the linear triplet CH_2_ wave function due to PP constraints are larger than those for CH_4_ and CH_3_. (We note that whereas the SCGVB descriptions of CH_3_ and CH_4_ both involve standard covalent bonds, the corresponding description of CH_2_ features a recoupled pair bond dyad,^31^ with strong coupling between the two bonds, and so some differences are to be expected for CH_2_.)

As is to be expected,^19^ the SCGVB description of CH_3_ features three predominantly singlet-coupled σ orbital pairs—(φ_1_, φ_2_), (φ_3_, φ_4_), and (φ_5_, φ_6_), with the odd-numbered orbitals being the C-based hybrids and the even-numbered ones being the corresponding H-based orbitals—with φ_7_ being based on C(2p_π_). For linear triplet CH_2_, the predominantly singlet-coupled σ orbital pairs are (φ_1_, φ_2_) and (φ_3_, φ_4_) (again with the odd-numbered orbitals being the C-based hybrids and the even-numbered ones being the corresponding H-based orbitals), with φ_5_ and φ_6_ being based on C(2p_π_) functions directed at 90° to one another. Just as in the case of CH_4_, the various C-based sp^*x*^-like hybrids (and C(2p_π_)-based functions) in CH_3_ and in CH_2_ arise in these calculations without any preconceptions. The weights in the Kotani basis for the modes of coupling with perfect pairing of the spins of the electrons occupying the σ orbitals are 0.9349 and 0.8969 for CH_3_ and linear CH_2_, respectively. Visual depictions of symmetry-unique SCGVB orbitals for CH_3_ and for linear CH_2_ are displayed in the second and third rows, respectively, of Figure 1. There are obvious similarities for φ_1_ and φ_2_ to the corresponding orbitals for CH_4_. As was the case for CH_4_, visual representations of the corresponding SCGVB(PP) and SCGVB(PP/SO) orbitals for CH_3_ and linear CH_2_ (available in the Supporting Information) are rather similar to those shown in Figure 1 for the ‘full’ SCGVB calculations.

We find that the various same-pair and different-pair orbital overlaps for CH_3_ vary in a similar fashion to those for CH_4_. The SCGVB same-pair (‘in-bond’) orbital overlap ⟨φ_1_ | φ_2_⟩ of 0.751 increases to 0.825 on restricting the mode of spin coupling to perfect pairing, with a further small increase to 0.838 on imposing the strong orthogonality constraint for the different pairs. At the same time, the restriction to perfect pairing reduces the SCGVB value of 0.464 for the different-pair overlap ⟨φ_1_ | φ_3_⟩ between C-based orbitals to 0.285. We observe from Table 1 that *h*_2p/2s_(φ_1_) for SCGVB(PP/SO) is almost the classical value of 2, but it drops to ∼0.9 for SCGVB(PP) and to ∼0.53 for ‘full’ SCGVB. Just as in the case of CH_4_, there is no reduction with decreasing *h*_2p/2s_(φ_1_) of the extent to which orbital φ_1_ can be considered a C-based hybrid: the values of *P*_2s+2p_^2^(φ_1_) for SCGVB, SCGVB(PP), and SCGVB(PP/SO) are 0.985, 0.978, and 0.970, respectively. Our estimates of the interatomic kinetic energy component for the three C–H bonds, *K*_CH_^σ^, are listed in Table 2 for CH_3_ and, as was the case for CH_4_, we find that *K*_CH_^σ^ varies monotonically with, but faster than, *E*_total_.

The symmetry-unique valence LNOs for CH_3_ generated using the SCGVB spinless one-particle density matrix are shown in the second row of Figure 2, with the corresponding pictures for CASSCF(7,7), SCGVB(PP) and SCGVB(PP/SO) being available in the Supporting Information. All in all, they are much as we should expect and, given what was described above for CH_4_, it comes as no surprise that we observe from Table 1 only small variations in *h*_2p/2s_(ψ_1_^LNO^) and rather similar values for the corresponding σ valence electron densities. All of these *h*_2p/2s_ values turn out to be close to 1.85.

As can already be seen from the values of *h*_2p/2s_(ψ_1_^LNO^) in Table 1, some aspects of our results for linear CH_2_ are slightly different from those for CH_4_ and CH_3_, even though it is still the case (see Table 2) that *K*_CH_^σ^ varies monotonically with, but faster than, *E*_total_. For this system, the overlap between the different C-based hybrids was in fact found to increase from SCGVB to SCGVB(PP), with the values of ⟨φ_1_ | φ_3_⟩ being 0.277 and 0.583, respectively. At the same time, the same-pair (‘in-bond’) overlap ⟨φ_1_ | φ_2_⟩ decreases slightly from 0.805 for SCGVB to 0.795 for SCGVB(PP) before increasing again to 0.849 for SCGVB(PP/SO). Consistently, *h*_2p/2s_(φ_1_) decreases from almost unity for SCGVB(PP/SO) to ∼0.56 for SCGVB but the corresponding value for SCGVB(PP) of ∼0.26 is not intermediate (see Table 1). As before, orbital φ_1_ can in each case be considered a C-based hybrid, given that the values of *P*_2s+2p_^2^(φ_1_) for SCGVB, SCGVB(PP), and SCGVB(PP/SO) are 0.992, 0.981, and 0.978, respectively.

Symmetry-unique valence LNOs for linear triplet CH_2_ are shown in the third row of Figure 2 (with the corresponding pictures for CASSCF(6,6), SCGVB(PP), and SCGVB(PP/SO) being available in the Supporting Information), and are again much as we should expect. Furthermore, there are no obvious signs of anything unusual about the LNOs for SCGVB(PP). We observe from Table 1 that the values of *h*_2p/2s_(ψ_1_^LNO^) and of those for the σ valence density of this system are all close to the classical value of unity, without any of the dramatic variations that are seen for the corresponding *h*_2p/2s_(φ_1_) values.

These various results for trigonal doublet CH_3_ (D_3*h*_) and for linear triplet CH_2_ (D_∞*h*_) indicate that our general findings for the notional sp^3^ case are not specific to ‘closed shell’ CH_4_ (T_*d*_) but that they also apply to systems that could be described classically in terms of sp^2^ and sp hybridization. A small caveat arising from the results for linear CH_2_ is that although the SCGVB(PP) total energy is necessarily intermediate between those for ‘full’ SCGVB and SCGVB(PP/SO), the SCGVB(PP) orbital overlaps and *h*_2p/2s_(φ_1_) values need not always be intermediate between those for SCGVB(PP/SO) and ‘full’ SCGVB. Nonetheless, the interatomic kinetic energy component for the bonds can still be expected to vary monotonically with, but faster than, the corresponding total energy.

## 4 Summary and Conclusions

Whereas the four classical sp^3^ hybrid atomic orbitals invoked^2,3^ in the traditional VB description of ‘closed shell’ CH_4_ (T_*d*_) are mutually orthogonal, the optimized orbitals that emerge from contemporary ab initio VB calculations turn out to have substantial overlaps with one another. These orbital overlaps are associated with increased C(2s) character in the individual orbitals, so that the 2p/2s ratio can be significantly less than the classical value of 3. For example, Shaik et al. used VBSCF orbital overlaps to deduce a p/s ratio for CH_4_ of 1.76.^7^ We found in the present study that the corresponding SCGVB value is even more extreme, namely 0.57, as was observed by Xu and Dunning,^5^ and we also reproduced their finding that the p/s ratio changes dramatically upon restricting the mode of spin coupling to perfect pairing (PP) and also upon applying the constraint of strong orthogonality (SO). Unsurprisingly, the orthogonality constraints in the SCGVB(PP/SO) calculations lead to orbitals with p/s ratios that are closest to the classical value. However, we must conclude from our analysis of total valence electron densities, and also of LNOs, that the p/s ratios for SCGVB orbitals, and likely VBSCF orbitals, do not provide a reasonable representation of the overall hybridization status of the carbon atom.

We observed somewhat analogous behavior for trigonal CH_3_ (D_3*h*_) and for triplet CH_2_ distorted from its ground state geometry so as to be linear (D_∞*h*_). Given that it is now well established that the formation of conventional covalent bonds involves the lowering of the interatomic kinetic energy,^22−28^ it is satisfying that we found that the relaxation of the constraints from SCGVB(PP/SO) to SCGVB(PP) to ‘full’ SCGVB leads in each case to an additional lowering of our estimates of the interatomic kinetic energy.

For the traditional description of CH_4_ based on classical orthogonal sp^3^ hybrids, the overall hybridization status of the C atom is also sp^3^, with analogous statements applying to sp^2^ hybrids in trigonal CH_3_ and to sp hybrids in linear triplet CH_2_. If instead the corresponding orbitals overlap one another, as is the case for contemporary ab initio VB calculations, then off-diagonal terms in the evaluation of the electron density have the consequence that the p/s ratio for the valence electron density is more likely than not to differ from that of individual orbitals. We demonstrated that the p/s ratios for localized natural orbitals generated by means of a particular isopycnic transformation, for which the evaluation of the density involves only diagonal terms, are far more consistent with the overall hybridization of the central atom.

For the three representative systems studied in the present study, we found that the overall hybridization status of the central atom exhibits only fairly small variations with the level of theory, both when non-dynamical and dynamical electron correlation effects are accounted for properly, and that the resulting values are somewhat closer to the classical ones. In the case of CH_4_, for example, we consistently observed values close to 2.7. As for the energetic impetus for the remaining differences from classical integer values of the p/s ratios for the total σ valence electron densities, we are drawn for the systems studied here to the ideas advanced by Shaik et al.^7^ of reductions in the promotion energy required to achieve maximum bonding, on account of enhanced C(2s) character.

## Supporting Information Available

Additional visual depictions of orbitals, as described in the text, and p/s ratios obtained using Mulliken population analysis (PDF)
