# Supplementary material for: Reassessing the Composition of Hybrid Orbitals in Contemporary VB Calculations
Source: J Phys Chem A. 2023 May 31;127(23):4949–56. doi: 10.1021/acs.jpca.3c01857 (PMC10278127; doi:10.1021/acs.jpca.3c01857)
Supplement: Supplementary file 1 — jp3c01857_si_001.pdf [file jp3c01857_si_001.pdf]

Supporting Information

Reassessing the Composition of Hybrid Orbitals in Contemporary VB Calculations

David L. Cooper[‡], Fabio E. Penotti[†] and Peter B. Karadakov[§]

[‡]*Department of Chemistry, University of Liverpool, Liverpool L69 7ZD, UK*

[†]*Consiglio Nazionale delle Ricerche, Istituto di Scienze e Tecnologie Chimiche "Giulio Natta", Via Golgi 19, I–20133 Milano MI, Italy*

[§]*Department of Chemistry, University of York, Heslington, York YO10 5DD, UK*

Figure S1

Depictions of symmetry-unique SCGVB(PP) orbitals for CH_4 (top row), CH_3 (middle row) and linear triplet CH_2 (bottom row).

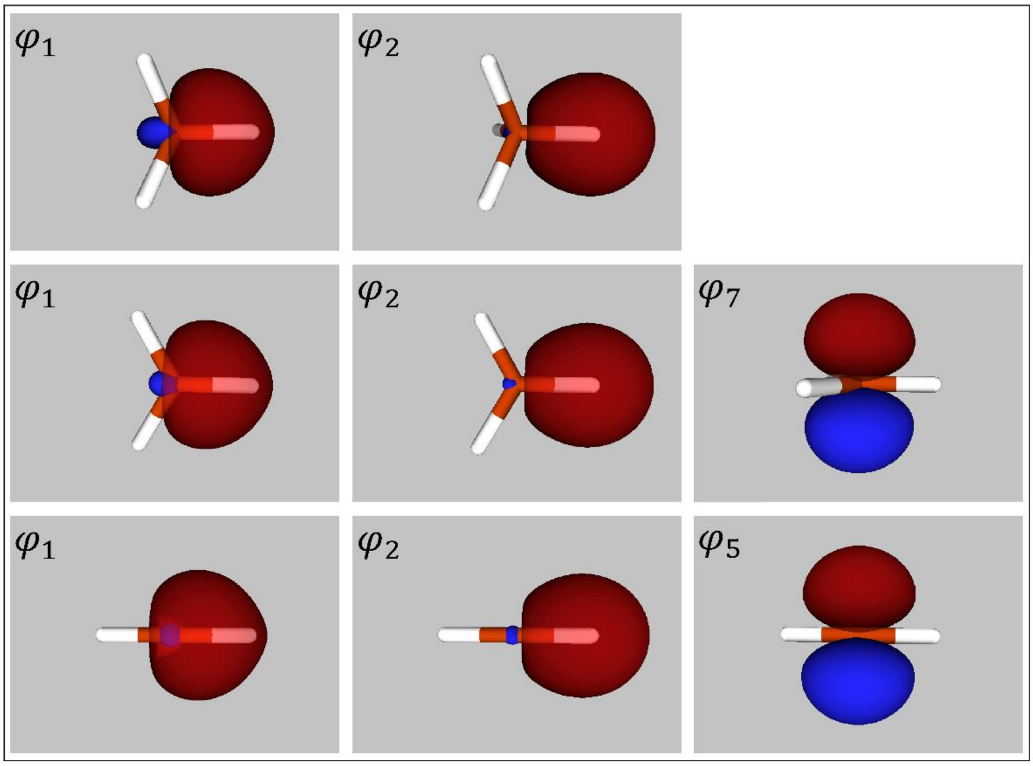

Figure S2

Depictions of symmetry-unique SCGVB(PP/SO) orbitals for CH_4 (top row), CH_3 (middle row) and linear triplet CH_2 (bottom row).

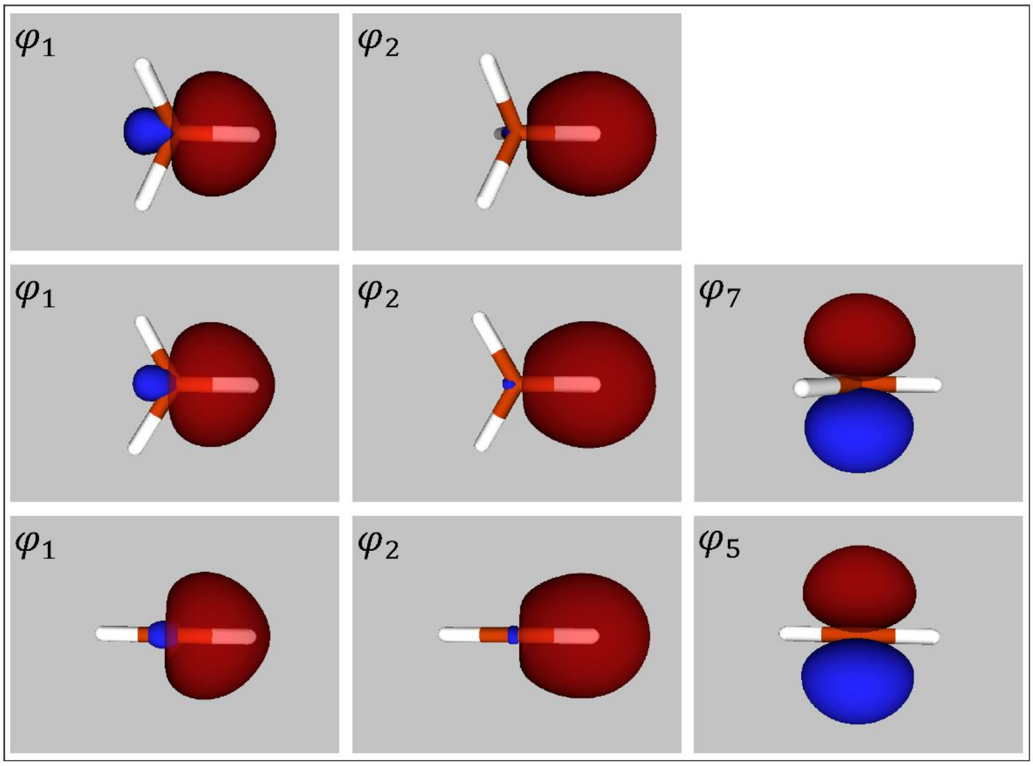

Figure S3

Depictions of symmetry-unique CASSCF(N,N) valence LNOs for CH_4 (top row), CH_3 (middle row) and linear triplet CH_2 (bottom row). Also shown are the corresponding occupation numbers, ν_i^{LNO}. The LNOs have been numbered in each case in order of decreasing ν_i^{LNO}.

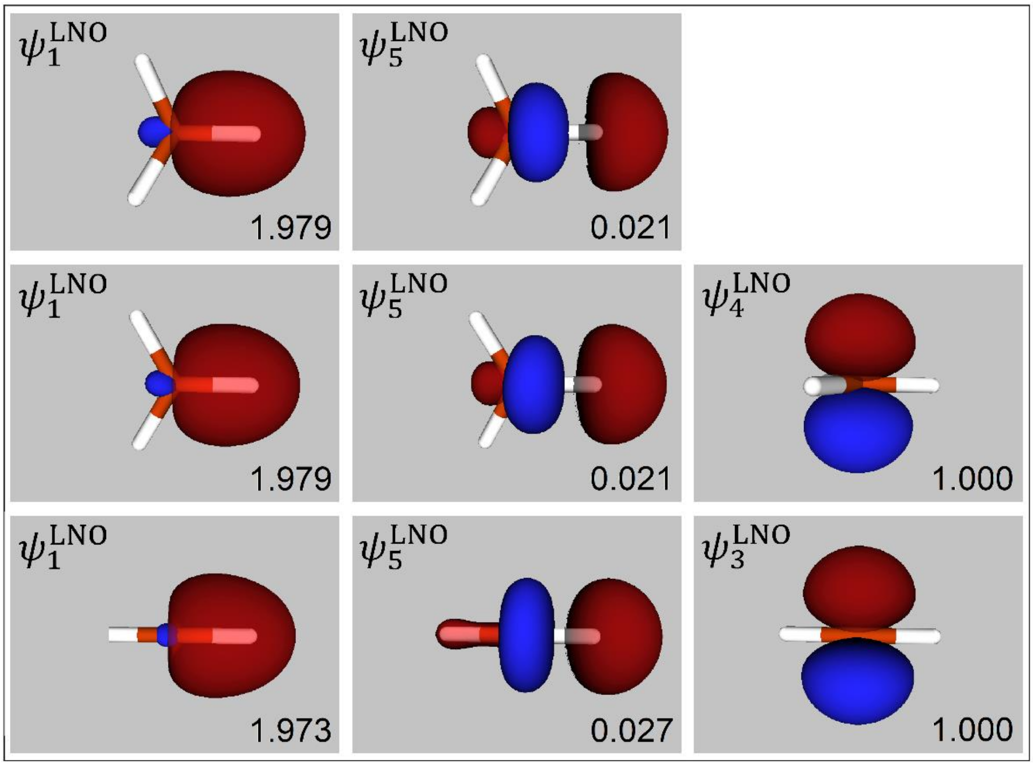

Figure S4

Depictions of the first two symmetry-unique valence LNOs for CH_4 from CCSD(fc) (top row) and *ic*-CASSCF(8,8)+1+2 (bottom row) densities. Also shown are the corresponding occupation numbers, ν_i^{LNO}. The LNOs have been numbered in order of decreasing ν_i^{LNO}.

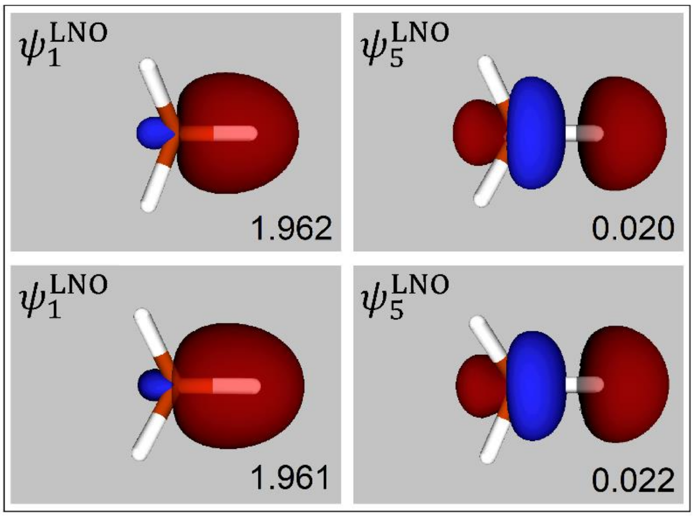

Figure S5

Depictions of symmetry-unique SCGVB(PP) valence LNOs for CH_4 (top row), CH_3 (middle row) and linear triplet CH_2 (bottom row). Also shown are the corresponding occupation numbers, ν_i^{LNO}. The LNOs have been numbered in each case in order of decreasing ν_i^{LNO}.

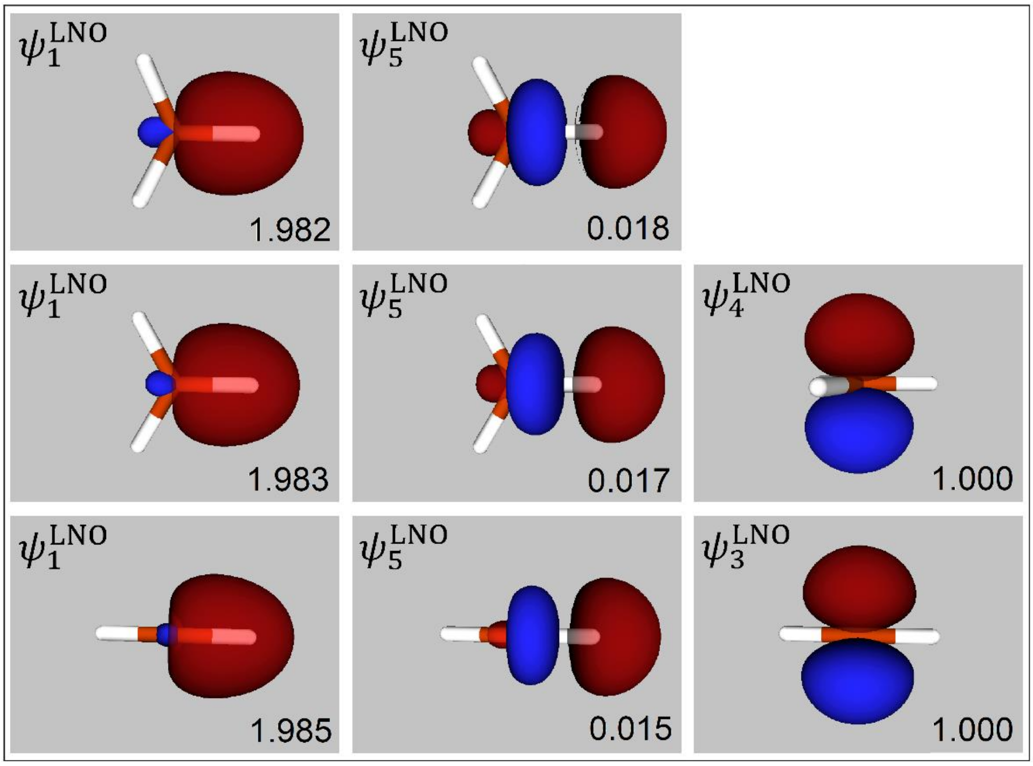

Figure S6

Depictions of symmetry-unique SCGVB(PP/SO) valence LNOs for CH_4 (top row), CH_3 (middle row) and linear triplet CH_2 (bottom row). Also shown are the corresponding occupation numbers, ν_i^{LNO}. The LNOs have been numbered in each case in order of decreasing ν_i^{LNO}.

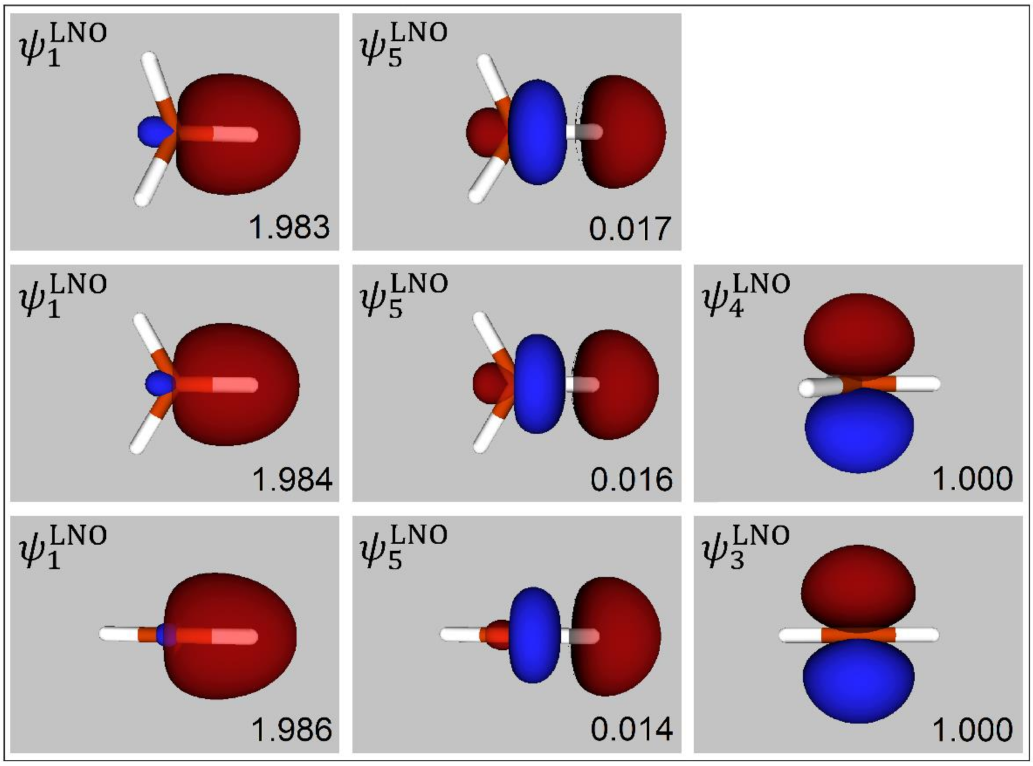

Table S1

Various p/s ratios ($h_{2p/2s}$) obtained using Mulliken population analysis for CH_4 (T_d), CH_3 (D_{3h}) and linear triplet CH_2 ($D_{\infty h}$).

	Level of theory	$h_{2p/2s}$		
		φ_1	ψ_1^{LNO}	σ valence density
CH_4	SCGVB(PP/SO)	2.21	2.71	2.72
	SCGVB(PP)	1.28	2.67	2.68
	SCGVB	0.53	2.68	2.69
	CASSCF(8,8)		2.69	2.70
	MRCI		2.68	2.69
	CCSD(fc) [a]		2.70	2.71
CH_3	SCGVB(PP/SO)	1.55	1.60	1.60
	SCGVB(PP)	0.77	1.57	1.58
	SCGVB	0.49	1.59	1.59
	CASSCF(7,7)		1.59	1.59
CH_2	SCGVB(PP/SO)	0.87	0.85	0.84
	SCGVB(PP)	0.28	0.83	0.82
	SCGVB	0.56	0.84	0.84
	CASSCF(6,6)		0.84	0.83

[a] Using r_{CH}=1.08598611 Å from full CCSD(T).

Full version of Ref. 14:

Werner, H.-J.; Knowles, P. J.; Knizia, G.; Manby, F. R.; Schütz, M.; Celani, P.; Györffy, W.; Kats, D.; Korona, T.; Lindh, R.; Mitrushenkov, A.; Rauhut, G.; Shamasundar, K. R.; Adler, T. B.; Amos, R. D.; Bennie, S. J.; Bernhardsson A.; Berning, A.; Cooper, D. L.; Deegan, M. J. O.; Dobbyn, A. J.; Eckert, F.; Goll, E.; Hampel, C.; Heßelmann, A.; Hetzer, G.; Hrenar, T.; Jansen, G.; Köppl, C.; Lee, S. R.; Liu, Y.; Lloyd, A. W.; Ma, Q.; Mata, R. A.; May, A. J.; McNicholas, S. J.; Meyer, W.; Miller III, T. F.; Mura, M. E.; Nicklass, A.; O'Neill, D. P.; Palmieri, P.; Peng, D.; Petrenko, T.; Pflüger, K.; Pitzer, R.; Reiher, M.; Shiozaki, T.; Stoll, H.; Stone, A. J.; Tarroni, R.; Thorsteinsson, T.; Wang, M.; Welborn, M., *MOLPRO, version 2022.2, a package of ab initio programs*. Cardiff, U. K., see https://www.molpro.net (accessed 20-Mar-2023).
